# Further Insight into Extractable (Organo)fluorine Mass Balance Analysis of Tap Water from Shanghai, China

**DOI:** 10.1021/acs.est.3c02718

**Published:** 2023-09-15

**Authors:** Enmiao Jiao, Pontus Larsson, Qi Wang, Zhiliang Zhu, Daqiang Yin, Anna Kärrman, Patrick van Hees, Patrik Karlsson, Yanling Qiu, Leo W. Y. Yeung

**Affiliations:** † Key Laboratory of Yangtze River Water Environment, College of Environmental Science and Engineering, Tongji University, Shanghai 200092, China; ‡ Shanghai Institute of Pollution Control and Ecological Security, Shanghai 200092, China; § Man-Technology-Environment Research Centre (MTM), School of Science and Technology, Örebro University, SE-70182 Örebro, Sweden; ∥ State Key Laboratory of Marine Pollution, City University of Hong Kong, Hong Kong 999077, China; ⊥ Eurofins Food and Feed Testing Sweden AB, Sjöhagsgatan 3, SE-531 40 Lidköping, Sweden

**Keywords:** extractable fluorine (EF), tetrafluoroborate (BF_4_^−^), hexafluorophosphate (PF_6_^−^), suspect screening, bis(trifluoromethanesulfonyl)imide (NTf_2_), ultra-short PFAS

## Abstract

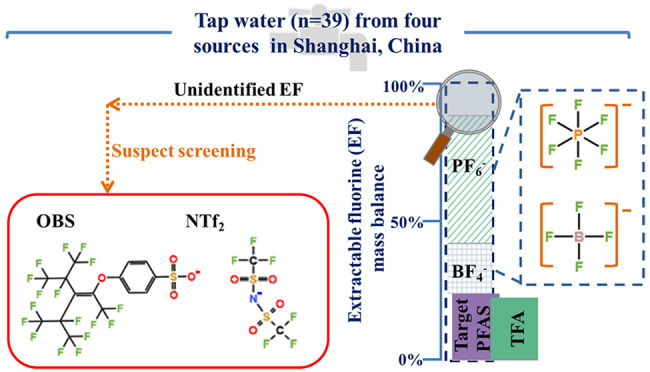

The ubiquitous occurrence of per- and polyfluoroalkyl substances (PFAS) and the detection of unexplained extractable organofluorine (EOF) in drinking water have raised growing concerns. A recent study reported the detection of inorganic fluorinated anions in German river systems, and therefore, in some samples, EOF may include some inorganic fluorinated anions. Thus, it might be more appropriate to use the term “extractable fluorine (EF) analysis” instead of the term EOF analysis. In this study, tap water samples (*n* = 39) from Shanghai were collected to assess the levels of EF/EOF, 35 target PFAS, two inorganic fluorinated anions (tetrafluoroborate (BF_4_^–^) and hexafluorophosphate (PF_6_^–^)), and novel PFAS through suspect screening and potential oxidizable precursors through oxidative conversion. The results showed that ultra-short PFAS were the largest contributors to target PFAS, accounting for up to 97% of ΣPFAS. To the best of our knowledge, this was the first time that bis(trifluoromethanesulfonyl)imide (NTf_2_) was reported in drinking water from China, and *p*-perfluorous nonenoxybenzenesulfonate (OBS) was also identified through suspect screening. Small amounts of precursors that can be oxidatively converted to PFCAs were noted after oxidative conversion. EF mass balance analysis revealed that target PFAS could only explain less than 36% of EF. However, the amounts of unexplained extractable fluorine were greatly reduced when BF_4_^–^ and PF_6_^–^ were included. These compounds further explained more than 44% of the EF, indicating the role of inorganic fluorinated anions in the mass balance analysis.

## Abstract 2

NTf_2_ was first reported in Chinese drinking water from Shanghai, and inorganic fluorinated anions contributed a significant proportion to EF mass balance analysis.

## 1 Introduction

Per- and polyfluoroalkyl substances (PFAS) are a group of anthropogenic fluorinated chemicals which contain at least one fully fluorinated methyl or methylene carbon atom.^1^ Due to their unique properties (e.g., stability and oil/water repellency), PFAS have extensive areas of use in diverse fields throughout society, such as textiles, paper and food packaging, fire-fighting foam, and household products.^2^ During the last 20 years, the ubiquitous presence of PFAS has resulted in the substances being found in humans,^3−5^ drinking water,^6−8^ food,^9,10^ and environmental compartments.^11−13^ PFAS have thus aroused particular attention due to their toxicity (e.g., metabolic disrupting effects, cardiovascular toxicity, and immunotoxicity), which has been demonstrated by a number of studies.^14−16^ Some PFAS have already been regulated under the Stockholm Convention. In addition to the already listed compounds (perfluorooctanoic acid (PFOA), its salts and PFOA-related compounds and perfluorooctane sulfonic acid (PFOS), its salts and perfluorooctane sulfonyl fluoride (PFOSF)), additional compounds (perfluorohexane sulfonic acid (PFHxS), its salts and PFHxS-related compounds) were also added to Annex A in 2022.^17^ The regulations, therefore, have led to the phasing out of these substances, and replacements (mainly the substitutes of PFOA and PFOS) have been produced for complementary applications. Toxicity studies of these emerging PFAS have discovered that some substitutes could have similar or even higher toxicity compared to their predecessors,^18^ suggesting that more attention needs to be given to various emerging PFAS.

Currently, targeted methods, such as liquid chromatography coupled to mass spectrometry, are the most commonly applied methods for the quantification of dozens of PFAS. However, the Organization for Economic Cooperation and Development (OECD) reported that there are approximately 5000 PFASs registered on the global market, and the new definition of PFAS also includes pesticides and pharmaceuticals (containing at least one −CF_2_– or −CF_3_ group), further increasing the number of compounds being defined as PFAS to more than 10 000. These new PFAS are not the focus of the PFAS analysis, but they may contribute to extractable organofluorine (EOF) and to oxidative conversion of precursor compounds if trifluoroacetic acid (TFA) is considered for PFCAs. The comprehensive characterization of PFAS in complex matrices is difficult to perform with targeted methods due to the lack of reference standards.^19^ Therefore, high-resolution mass spectrometry (HRMS) techniques (e.g., quadrupole-time-of-flight mass spectrometry (QTOF-MS), Fourier transform ion cyclotron resonance MS and Orbitrap MS)) are employed to identify novel PFAS.^20−22^ Methods using oxidative conversion of precursor compounds (e.g., total oxidizable precursor (TOP) assay and the use of photocatalyst) have also been developed to investigate the presence of potential oxidizable precursors in samples.^23,24^ However, HRMS is mainly applied to identify new substances rather than for quantification, while oxidative conversion is mostly used for quantification rather than for identification.

To obtain information that is overlooked from targeted analysis, various techniques are currently available to help expand the analytical range (e.g., total fluorine analysis, adsorbable organofluorine analysis (AOF) and EOF analysis using combustion ion chromatography (CIC), particle-induced gamma-ray emission spectrometry (PIGE), and continuum source molecular absorption spectrometry (CS-MAS)).^12,25−27^ Among these techniques, EOF-based mass balance analysis has been widely used to elucidate the fraction of unexplained EOF through mass balance analysis (i.e., levels of EOF minus levels of known target PFAS).^28^ Previous studies have discovered a high percentage of unexplained EOF in drinking water and other water matrices.^12,29^ The extraction methods used in these studies were based on solid phase extraction (SPE), using weak anion exchange (WAX) with an additional washing step to remove the free fluoride. A recent study, which used a similar extraction method,^30^ reported the detection of inorganic fluorinated anions in German river systems, suggesting that unexplained EOF may also consist of inorganic fluorinated anions that are not removed during sample treatment. These identified inorganic fluorinated anions belong to ionic liquids or electrolyte salts used in lithium-ion batteries, for example, tetrafluoroborate (BF_4_^–^) and hexafluorophosphate (PF_6_^–^).^31,32^ Therefore, it may be more appropriate to use the term “extractable fluorine (EF) mass balance and unexplained EF” instead of EOF or unexplained EOF if they were detected in the EOF fraction of the sample.

Shanghai, located in the estuary of the Yangtze River, is one of the most economically developed regions in China. The four reservoirs in Shanghai (Figure S1) provide drinking water to more than 20 million people. However, with rapid urbanization and the development of various industries, these water sources may be vulnerable to pollution. Drinking water is an important human exposure route, and it has already been shown that levels of PFAS in human serum are related to those in drinking water.^33^ A number of studies focusing on the ubiquitous presence of PFAS in Chinese drinking water have been conducted.^34−36^ The standard for drinking water quality in China was also published in 2022, which included PFOA (80 ng/L) and PFOS (40 ng/L), indicating the importance of PFAS monitoring for drinking water safety. However, most studies focused on the routinely monitored PFAS, and more attention needs to be given to the expansion of the analytical range to obtain a more comprehensive picture.

The objectives of this study are (1) to conduct an EF/EOF mass balance analysis in tap water collected from Shanghai by measuring the concentrations of EF/EOF, target PFAS (including ultra-short PFAS having less than four carbon atoms), and inorganic fluorinated anions (BF_4_^–^ and PF_6_^–^); (2) to perform suspect screening and oxidative conversion to investigate the potential contribution of these substances to unexplained fractions; and (3) to conduct a comparison with available guidelines. Additionally, the inorganic fluorinated anions were also analyzed in tap water samples collected from Sweden (*n* = 3), Denmark (*n* = 1), and Norway (*n* = 2) for comparison.

## 2 Materials and Methods

### 2.1 Chemicals

Detailed information on chemicals is provided in the Supporting Information.

### 2.2 Sample Collection and Solid Phase Extraction (SPE) for Water Samples

A total of 39 tap water samples were collected in July 2021 and the sampling sites are presented in Figure S1. Sampling locations covered all of the drinking water sources in Shanghai, including tap water whose source water is from reservoirs A (*n* = 7), B (*n* = 2), C (*n* = 10), and D (*n* = 20). Tap water samples were collected from Sweden (*n* = 3), Denmark (*n* = 1), and Norway (*n* = 2) in 2022. All samples were kept at 4 °C until analysis.

Tap water samples from Shanghai were extracted in triplicate: replicate 1 (500 mL for quantifying most of the PFAS) and replicate 2 (50 mL only for quantifying the ultra-short PFAS) were spiked with internal standards (IS) before extraction and were used for target analysis; replicate 3 (1 L) was extracted without spiking any IS before extraction and was used for EF/EOF analysis. Extractions were conducted using a solid phase extraction (SPE) method using weak anion exchange (WAX) cartridges^12,28^ according to the workflow (Figure S2) given in the Supporting Information. Extraction of tap water samples collected from Sweden, Denmark, and Norway followed the EF/EOF analysis and was only used for the comparison of inorganic fluorinated anions.

### 2.3 Oxidative Conversion of Precursors

The oxidative conversion of precursor compounds was similar to the TOP assay, but the oxidation was performed on sample extracts. The method was modified based on the previous method (increased doses in both oxidant and base) to ensure complete reaction.^23,37^ The extracts were evaporated to near dryness and then diluted with 750 μL of 10 M NaOH solution and 25 mL of 120 mM K_2_S_2_O_8_ solution. The solutions were then placed into a preheated water bath (85 °C) for 6 h. After the reaction, the solutions were cooled down to room temperature before the pH was adjusted to 2 using formic acid. After oxidative conversion, the extraction of the samples followed a similar extraction procedure as target analysis but with a larger amount of sorbent material and larger particle size (500 mg, 60 μm). In addition, the washing step of 20 mL of ultrapure water with 0.01% NH_4_OH was skipped to improve the recovery of TFA.

### 2.4 Instrumental Analysis for Target PFAS, BF_4_^–^, PF_6_^–^, and EF/EOF

The ultra-short PFAS, BF_4_^–^, and PF_6_^–^ were quantified using a supercritical fluid chromatograph (SFC, Acquity Ultra Performance Convergence Chromatograph, Waters Corporation, Milford, MA, USA) coupled to a tandem mass spectrometer (MS/MS; Xevo TQ-S, Waters Corporation, Milford, MA, USA) operated in electrospray negative ionization (ESI−) mode. Chromatographic separation was achieved using a Torous DIOL column (3 mm × 150 mm, 1.7 μm; Waters Corporation Milford, USA), which was maintained at 50 °C. Supercritical CO_2_ and methanol with 0.1% NH_4_OH were used as the mobile phase. Other parameters are reported elsewhere.^38^ Unlike ultra-short PFAS, monitoring of BF_4_^–^ and PF_6_^–^ was based on single ion monitoring due to nondetectable fragments of these small ions. In this case, samples and standards were analyzed on SFC-MS/MS using three analytical columns (DIOL, BEH, and HSS C18 SB). The results showed that the retention time in samples matched standards in the three columns. Moreover, the isotopic patterns of BF_4_^–^ were found to match to natural isotopic distribution which further supported the identification of BF_4_^–^. Further details of the identification of BF_4_^–^ and PF_6_^–^ are provided in the SI.

For the majority of target PFAS, ultra performance liquid chromatography electrospray ionization tandem mass spectrometry (UPLC-MS/MS, Waters Corporation, Milford, MA, USA) in ESI– mode and an Acquity BEH C18 column (2.1 mm × 100 mm, 1.7 μm; Waters Corporation Milford, USA) were used. The mobile phase consisted of 2 mM NH_4_Ac in MeOH and 2 mM NH_4_Ac in 30:70 MeOH/ultrapure water. The gradient conditions were applied, and more details are provided in a previously published paper.^38^

Levels of EF/EOF were measured by combustion ion chromatography (CIC) according to a published method.^29^ In brief, 100 μL of sample extracts was combusted at 1000–1050 °C in the presence of water. The fluorine in the samples was converted into hydrogen fluoride and subsequently absorbed into ultrapure water. Two milliliters of the absorber solution was injected onto an ion exchange column (Metrosep A Supp5, 4 mm × 150 mm), which was used to separate the anions. The eluent used was a carbonate buffer (64 mM sodium carbonate and 20 mM sodium bicarbonate). After separation, the fluoride concentration was measured by the ion conductivity. Detailed information is described elsewhere.^39^

### 2.5 Suspect Screening

Four separate pooled samples were made by taking equal volumes of water samples from the same reservoir (finally a total of 1 L for each) and were extracted. Suspect screening analysis was performed by an Acquity UPLC system coupled to a quadrupole time-of-flight mass spectrometer (G2-XS, Waters Corporation, Milford, USA) in the electrospray negative ionization (ESI−) mode. An Acquity BEH C18 column (2.1 mm × 100 mm; 1.7 μm; Waters Corp., Milford, USA) was used for chromatographic separation. Mobile phases were the same as those for target analysis. A data independent acquisition mode (MS^E^) was performed in full scan (*m*/*z* 50–1200). Parameters and other details referred to previously published papers.^29,40^

Databases collected from the U.S. EPA CompTox Chemistry Dashboard, OECD, and other studies^41−43^ were used. The UNIFI software (Waters, Milford, USA) and R scripts (R, v 3.6.2; R Foundation for Statistical Computing: Vienna, Austria) were used for data processing. The following parameters were set for the initial setting to increase the confidence level: (1) signal to noise, >3; (2) LC peak width, less than 30 s; (3) intensity, >5 times the intensity in the extraction blank; and (4) mass error, less than 5 ppm.

### 2.6 Quality Assurance and Quality Control

For target analysis, two extraction blank samples and one recovery sample were prepared using ultrapure water in the lab for every batch to keep track of contamination or the loss of PFAS during the whole extraction. Isotopically labeled internal standards (IS, 2 ng) were added to all of the samples before extraction (including extraction blank samples, recovery samples, and real water samples), and additional native standards (calibration standard (CS, 2 ng)) were spiked in the recovery samples. All extraction blank samples and recovery samples followed the same extraction methods and were stored in the same way as the real water samples. After extraction procedures, isotopically labeled recovery standards (RS, 2 ng) were spiked into the sample prior to instrumental analysis to evaluate sample recoveries. The results of recovery samples are shown in Table S3; the recoveries ranged from 33% to 119%. The homogeneously mixed water sample was prepared by pooling equal proportions of the real water samples to obtain a bulk sample and then divided into several subsamples; they were used as quality control (QC) samples to show repeatability among different batches of extractions. In every batch of extraction, one of the subsamples was extracted in the same way as the real water samples. The relative standard deviation (RSD) of individual native PFAS in QC samples ranged from 5 to 24%; details of the results are provided in Table S4. PFAS were quantified using an internal calibration method with respective native standards and isotopically labeled standards. For those compounds where no corresponding isotopically labeled standard was available, the IS with the closest retention time from the same class was selected (Table S2). The recoveries of IS in real water samples are listed in Table S5. The average concentrations in extraction blanks plus 3 times the standard deviation were used as the method detection limit (MDL) and the average concentrations plus 10 times the standard deviation were used as the method quantification limit (MQL). For PFAS which showed no detection in extraction blanks, the lowest calibration curve point was used as the MQL (Table S7).

For EF/EOF analysis, two blank extraction samples and one QC sample were extracted for every batch to monitor EF/EOF contamination and the repeatability of EF/EOF analysis. PFOA standard at 250 ng/mL was injected in triplicate in the beginning of the sample analysis and was injected every nine samples during instrumental analysis; the RSDs of the triplicate injection of the standard and every nine samples were found to be 12% and 16%, respectively. The relative standard deviation of the measured EF/EOF in the QC samples was 35% (*n* = 7). The field blanks showed no detectable levels of EF/EOF. An external calibration curve of PFOA (ranging from 50 to 1000 ng/mL F equivalents) was used for quantification of EF/EOF in the CIC.^44^ The combustion blank levels, which represented the background of the instrument, were obtained when an empty quartz boat was combusted. Combustion blanks were analyzed between samples to avoid carry-over. If the extraction blank was found to be higher than 50 ng of F/mL (the lowest point on the calibration curve), MDL was then calculated with the average concentrations in extraction blanks plus 3 times the standard deviation in every batch of extraction. If the extraction blank was found to be lower than 50 ng of F/mL, the limit of 50 ng of F/mL was used. The MDL in this study ranged from 25 to 43.5 ng F/L after application of a concentration factor of 2000. As for the quantification of BF_4_^–^ and PF_6_^–^, their concentrations were quantified using an external calibration curve. The reported concentrations for BF_4_^–^ and PF_6_^–^ reflect the concentrations in the sample extract for EF mass balance analysis, not necessarily representing the levels in the water samples as the extraction method was not optimized for these compounds, and no spike recovery test had been performed.

For oxidative conversion of precursors, procedure blanks, one positive control sample (oxidant and base solutions spiked with 6:2 FTSA) and one negative control sample (ultrapure water spiked with 6:2 FTSA without adding any oxidant or base), were analyzed together with samples to monitor oxidation reactions and to keep track of contamination. Isotopically labeled FOSA was spiked in extracts before evaporating the solvent for oxidative conversion. The molar ratio of isotopically labeled FOSA to isotopically labeled PFOA was calculated to evaluate the reactions, and the average ratio was 88% (RSD: 36%). IS was also spiked before extraction, and the recoveries are presented in Table S6.

For suspect screening analysis, extraction blanks and batch standards were also analyzed together with samples, and only novel PFAS that were not identified in blanks and standards were selected. Considering the lack of standards for the PFAS identified through suspect screening, semiquantification was conducted using surrogate native standards (Table S10).

### 2.7 Mass Balance Analysis

To conduct a mass balance analysis, the measured concentrations of target PFAS need to be converted to fluorine-equivalent concentrations. Details are provided in the Supporting Information.

## 3 Results and Discussions

### 3.1 Occurrence of Target PFAS in Tap Water from Different Water Sources

**Figure 1:**
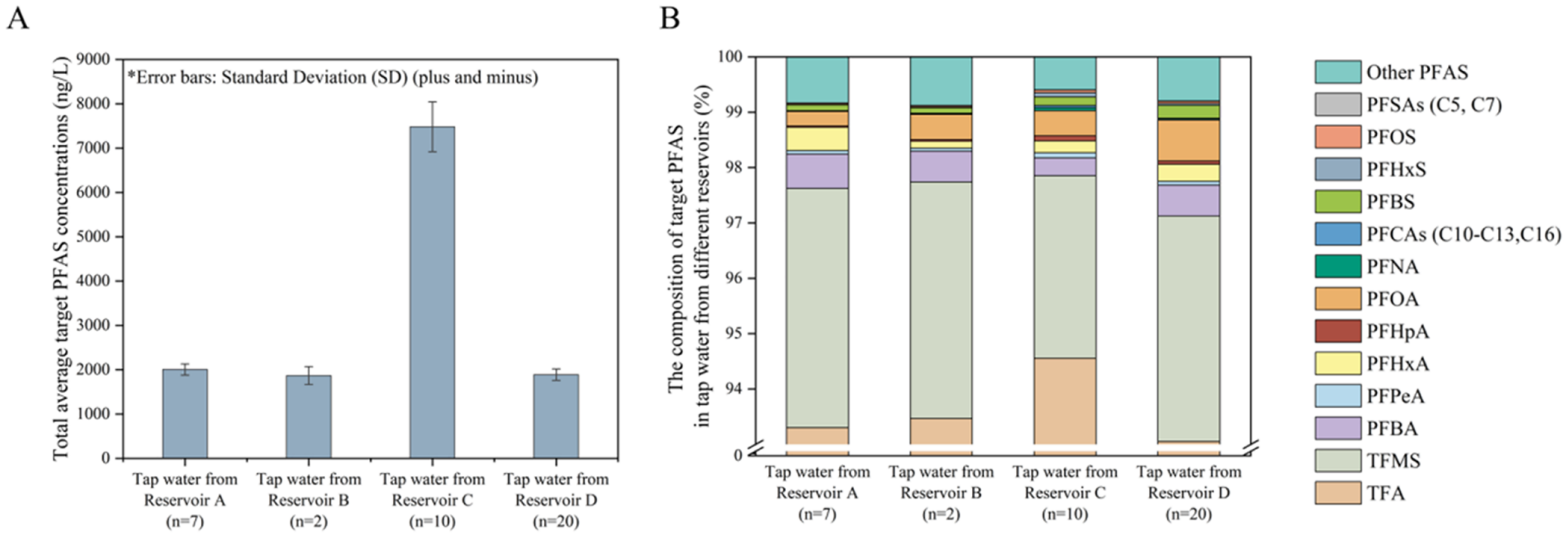
Total average target PFAS concentrations (A) and composition in tap water from different reservoirs (B). (A list of target PFAS is provided on the legend next to panel B.)

Among the 35 investigated target PFAS, 23 PFAS were detected at least once above MQL, and the results are summarized in Figure 1, Figure S6, and Table S8. The total PFAS concentrations in tap water from reservoirs A, B, C and D were in the range of 1850–2210, 1630–1970, 6630–8480, and 1490–2150 ng/L, respectively. Observable higher PFAS concentrations were detected in tap water from reservoir C, which could be explained by the presence of possible point sources in the area. There are high-density point sources, such as chemical industries and textile industries, close to Taipu River, which connect to reservoir C, Tai Lake and Huangpu River,^45,46^ which could be potential contributors to the high EF concentrations in tap water from reservoir C. The compositions of different PFAS decreased in the order of ultra-short PFAS (<C4, 98 ± 0.42%) > PFCAs (≥C4, 1.53 ± 0.34%) > PFSAs (≥C4, 0.26 ± 0.10%) > other PFAS (0.03 ± 0.02%). TFA was the largest contributor, accounting for more than 91% of ΣPFAS in all samples, with average concentrations ranging from 1.74 to 7.08 μg/L, which was on the same order of magnitude as a previous study on TFA in tap water from Shanghai.^47^ One known source of diffuse contamination of TFA is the transformation of hydrofluorocarbons and hydrochlorofluorocarbons.^38,48^ Additionally, the degradation of pharmaceuticals and emissions from industries or wastewater treatment plants can also yield TFA.^49^ Trifluoromethanesulfonic acid (TFMS) contributed to another 3.94% of total concentrations on average. PFPrA (average: 16.2 ng/L (A), 15.0 ng/L (B), 40.5 ng/L (C), and 14.6 ng/L (D)) was also prevalent with the sum concentrations accounting for 0.72% of ΣPFAS on average. Although PFPrA could be formed through the oxidation of some precursors,^50^ and TFMS was found to be related to organic synthesis and lithium-ion batteries,^51,52^ sources of these two ultra-short PFAS still remain largely unknown. Two infrequently detected ultra-short PFAS (perfluoroethanesulfonic acid (PFEtS) and perfluoropropanesulfonic acid (PFPrS)) showed no detectable levels in this study. They were previously reported in groundwater related to military training sites.^53^ A similar finding was also reported in another study, where TFA was found to be ubiquitous in sources of German drinking water (98% of ΣPFAS).^54^

PFCAs (≥C4) were another abundant group, with average sum concentrations in tap water from reservoirs A, B, C and D of 28, 21, 94, and 33 ng/L, respectively. The PFCA group patterns were dominated by PFBA, PFHxA, and PFOA, with the sum concentrations of the three compounds accounting for more than 77% of ΣPFCAs. Another study investigated PFAS in tap water from Shanghai and found that PFBA was the most dominant congener.^55^ The contribution of PFSAs and other PFAS was less than that of PFCAs, and these substances were only detected in tap water from reservoirs A, B, C, and D at 3.25, 2.51, 25.2, and 6.32 ng/L, respectively. PFBS was the dominant compound in the PFSA group, followed by PFOS in tap water from reservoirs A, B, and D, with the sum concentrations accounting for more than 85% of ΣPFSAs. In addition to PFBS and PFOS, the patterns of PFSAs were also dominated by PFHxS in tap water from reservoir C, and the three compounds contributed more than 98% of ΣPFSAs.

### 3.2 Extractable (Organo)fluorine (EF/EOF) Mass Balance Analysis

**Figure 2:**
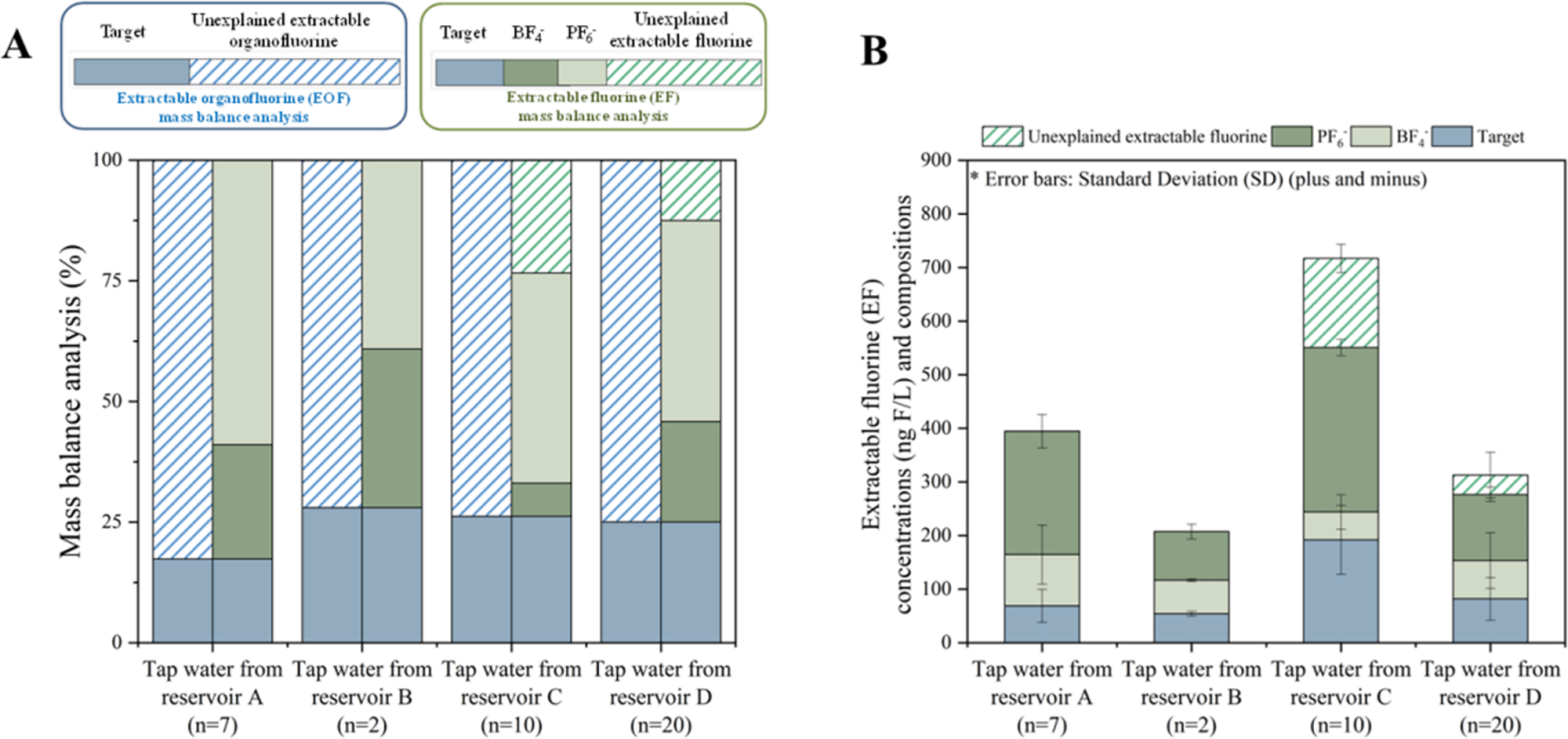
Extractable organofluorine (EOF) and extractable fluorine (EF) mass balance analysis (A) and extractable fluorine (EF) concentrations (ng F/L) and compositions (B) of tap water samples from reservoirs A, B, C, and D.

All samples showed detectable levels of EOF in this study, and a higher fraction of unexplained EOF (>64%) was found for tap water samples based on the mass balance approach (Figure 2A). For the unexplained EOF, novel PFAS and fluorinated pharmaceuticals could be potential sources.^42,56^ Inorganic fluorinated anions (BF_4_^–^ and PF_6_^–^) are another possible source, as suggested in a recent study.^30^ Results showed that BF_4_^–^ and PF_6_^–^ were detected in our water samples (Figure 2B); therefore, the term “extractable fluorine (EF) mass balance” was used.

An overview of EF concentrations and mass balance analysis is presented in Figure 2 and Table S9. A total of 23 out of 35 target PFAS and two forms of inorganic fluorinated anions (BF_4_^–^ and PF_6_^–^) were detected (Table S9). The highest average EF concentrations were detected in tap water from reservoir C (mean ± standard deviation; 717 ± 114 ng F/L). Similar mean EF concentrations were observed for tap water from reservoirs A, B, and D (382 ± 86, 199 ± 55, and 313 ± 87 ng F/L, respectively). Relatively high EF levels in tap water from reservoir C were observed, which was consistent with the results from the target analysis.

For target PFAS, the contribution of ultra-short PFAS (<C4) to EF was relatively large, accounting for 6–30% of EF. PFCAs (≥C4) accounted for a further 3–10% of EF. After subtracting the levels of target PFAS from EF, the amounts of unexplained extractable fluorine were revealed. Up to 64% of the EF was unexplained, indicating that PFAS contamination in the samples might be underestimated if only target PFAS were investigated. This is also consistent with what has been observed in drinking water treatment plants around Tai Lake, where more than 68% of EOF could not be explained by target PFAS.^29^

An additional washing step has been shown to remove over 99.995% of the free fluoride in the sample.^28^ However, BF_4_^–^ (9.3–195 ng/L) and PF_6_^–^ (93–391 ng/L) were detected at relatively high concentrations when compared with target PFAS (except for TFA) in this study. BF_4_^–^ accounted for 3–40% of EF, and the proportion of PF_6_^–^ to EF ranged from 24% to 82%. Taking BF_4_^–^ and PF_6_^–^ into account, the average EF mass balance could be closed in tap water from reservoirs A and B. In tap water from reservoirs C and D, the proportions of unexplained extractable fluorine were also largely reduced (to 23% and 12% on average, respectively). However, BF_4_^–^ and PF_6_^–^ were not detected in tap water collected from Sweden, Denmark, and Norway, which suggests that the occurrence of these compounds is source specific.

### 3.3 Suspect Screening and Oxidative Conversion of Precursors

Tap water samples were pooled together according to the source reservoir and extracted, and suspect screening was performed to identify novel PFAS. An additional eight PFAS (classes 1–5, Table S10) were matched to the databases after checking exact mass, fragments, and retention time through the suspect screening workflow. The classes, along with the MS2 information, are summarized in Table S10. Confidence levels were assigned based on the scale proposed by Schymanski et al.,^57^ and all of the classes were assigned to a confidence level of 3 or above.

Bis(trifluoromethanesulfonyl)imide (NTf_2_, class 1) was identified in all of the samples at a relatively high response. To the best of our knowledge, this is the first report of the presence of NTf_2_ in drinking water from China. NTf_2_ is widely used in ionic liquids and lithium-ion batteries and has recently been detected in river systems and sources of drinking water in Germany.^30,32,54^ NTf_2_ was found to have toxic effects in aquatic organisms and sludge bacteria,^58,59^ but toxicology studies are still limited. Further investigation on other aquatic organisms is needed.

Sodium *p*-perfluorous nonenoxybenzenesulfonate (OBS, class 2) was also ubiquitously detected as it was widely used as a coformulant in fire-fighting foam and the oil production field^60^ in China. OBS was previously reported in wastewater and surface water close to oilfields.^42,61^ One study also detected OBS in a DWTP at concentrations below 2 ng/L during the wet season.^62^

Among hydrosubstituted perfluoroalkyl ether sulfonate (H-PFECAs, class 3), two homologues (C4 and C8) were identified. The identification of 6:2 H-PFESA (C8) was not unexpected, as it is known to be the major transformation product of F-53B.^63^ Perfluoroalkyl sulfonamide (PFSM, class 4) and hydro substituted PFCAs (H-PFCAs (C10), class 5) were also identified in samples.

Total semiquantification concentrations of the eight novel PFAS (0.34–1.01 ng/L) only contributed to an addition of less than 0.10%. Although concentrations of these novel PFAS (identified through suspect screening) are low and did not explain much of the EF, further attention still needs to be paid to these PFAS, as most of the compounds have already been identified in the general population, indicating that humans are exposed to various novel PFAS and potential toxic effects may arise.^64^

In addition to suspect screening, oxidative conversion is also a useful tool to indicate the potential presence of unknown oxidizable precursors. The presence of unknown oxidizable precursors might be reflected in increased PFCA concentrations after oxidative conversion. In the pooled samples from each reservoir, TFA showed increases in pooled samples from reservoirs A (50.4 ng/L, 24%) and B (47.5 ng/L, 26%), but this only further explained less than 10% of the EF on average. Increases were also observed for other compounds (e.g., PFPrA, PFBA, and PFPeA, 24–28%) in some samples, but they only contributed to less than 1% of the EF. Hence, the results showed that unknown oxidizable precursors were of minor importance, demonstrating that oxidizable precursors were not prevalent in the samples in this study and did not make significant contributions to unknown fractions.

### 3.4 Comparison with Standards/Guideline Values and Estimation of Mixture Exposures

**Figure 3:**
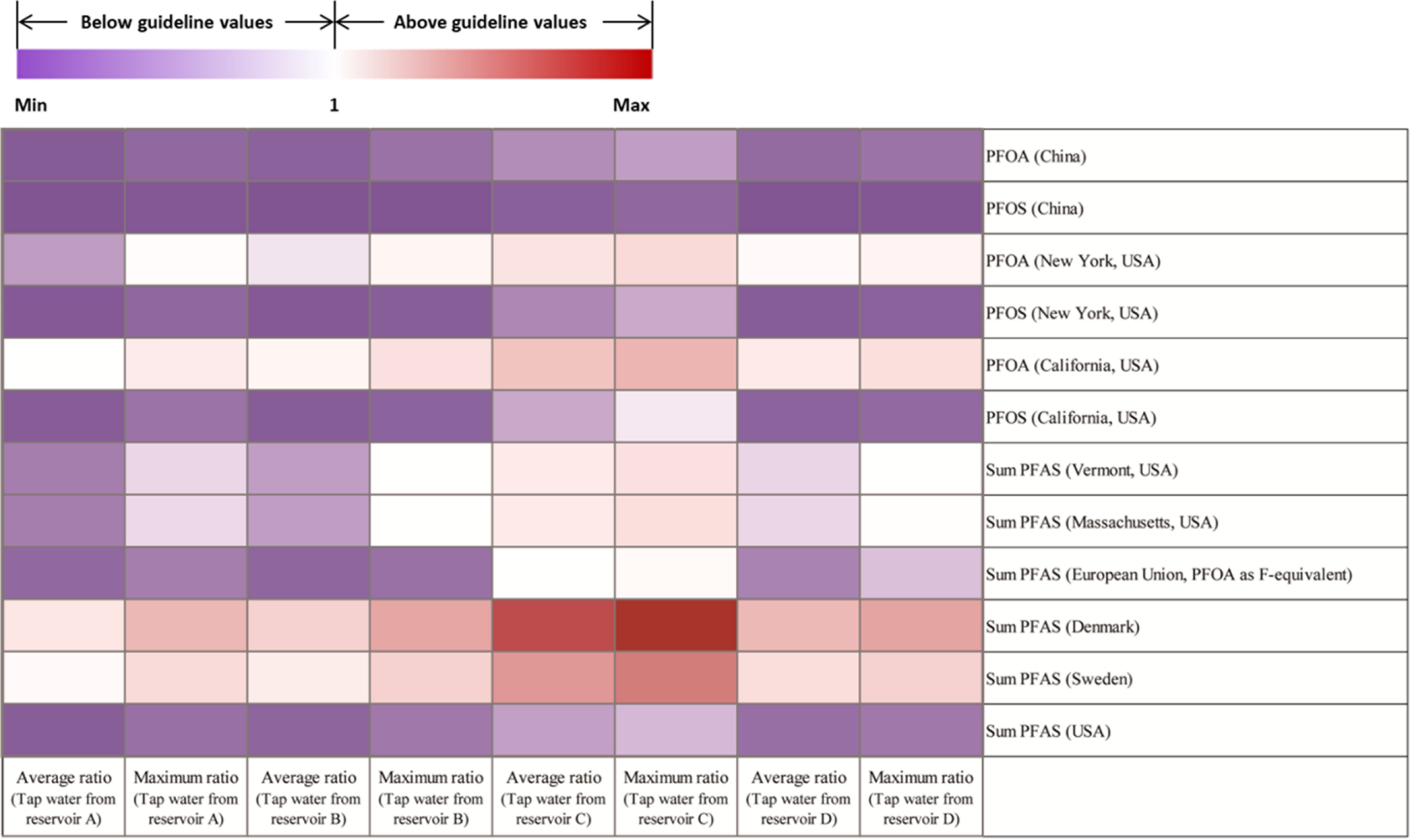
Ratios of drinking water from four reservoirs to different guideline values.

Ratios of average (maximum) measured PFAS concentrations with respect to standard/guideline values of PFAS in drinking water from different authorities were calculated to obtain a preliminary comparison. Stringent values for individual or combined PFAS from standards or guidance and advisory were selected,^65−69^ and the details such as whether they are promulgated are presented in Table S11 and Figure 3. PFAS in all of the samples did not exceed the Chinese standards for drinking water quality of 80 ng/L for PFOA and 40 ng/L for PFOS. However, high ratios (>1) were observed under stringent values issued by other countries, especially for samples from reservoir C. Compared to the guideline values issued by Denmark of 2 ng/L for four combined PFAS, drinking water in this study had PFAS exceeding the value, with the average (maximum) ratios ranging from 2.96 to 23.69 (7.96–32.04). Notably, the drinking water directive (500 ng/L for total PFAS) proposed by the European Union was also used to better estimate the overall status derived from total PFAS. Since the presence of BF_4_^–^ and PF_6_^–^ was already confirmed in EF mass balance analysis, the total PFAS estimation was conducted after subtracting BF_4_^–^ and PF_6_^–^ from EF. In a total PFAS estimation, EF concentrations were converted to total PFAS concentrations using PFOA as F-equivalent (conversion of PFOA to F-equivalent is around 69%). Compared with the total PFAS guideline value, the average (maximum) ratios for tap water from reservoirs A, B, C, and D were 0.16 (0.32), 0.13 (0.22), 1.04 (1.52), and 0.34 (0.73), respectively, indicating that tap water from reservoir C exceeded the total PFAS directive.

However, PFAS often exist together in exposure media, and the potential health concerns of mixture exposure may not be well represented if only ratios between PFAS concentrations and guideline values were calculated. The relative potency factor (RPF) methodology was therefore introduced in this study to help assess PFAS mixture exposure.^70^ In brief, RPFs of different PFAS were derived based on the analysis of the toxicity database retrieved from other literature, and the sum of PFOA equivalents in a mixture could be obtained by applying RPFs to individual PFAS quantities. The average individual PFAS concentrations in tap water from different reservoirs were used, and perfluorooctanoic acid equivalent (PEQ) was calculated based on RPFs (Table S12). The PEQs of different PFAS are illustrated in Figure S7. The sum of PEQ reflected the cumulative PFAS concentrations in tap water from reservoirs A, B, C and D, ranging from 9.49 to 10.63, 14.21 to 15.13, 99.73 to 119.38, and 21.35 to 23.22 ng/L, respectively. Compared with the Chinese standards, the sum of PEQ in tap water from reservoir C clearly exceeded the limit (80 ng/L), while levels in other tap water samples were all below 80 ng/L. When compared with the lowest PFOA guideline value (California, USA), however, the sum of PEQ in all tap water samples exceeded the value (5.1 ng/L), indicating the potential health concerns presented by exposure to PFAS mixtures through drinking water. Nevertheless, due to uncertainty related to additive PFOA-like effects in vivo following PFAS mixture exposure, the RPF result may have certain inaccuracies, and a more refined PFAS RPF approach is needed.

Overall, the results showed that PFAS in tap water from Shanghai exceeded certain stringent guideline values, and there is therefore an urgent need to improve the management and control measures.

## 4 Environmental Implications

Due to the difficulties of analyzing the thousands of PFAS, various studies have started to evaluate whether EOF can be a better option to reflect “total PFAS”. Free fluoride can be removed by the additional washing step to avoid its contribution to EOF analysis. However, the additional washing step was shown to result in poor recovery of TFA. As shown in this study, other forms of inorganic fluorinated anions were also retained during extraction, and their presence might influence EOF analysis or another similar nonspecific approach such as AOF, which might result in overestimating the OF in the sample extract. Our results show that these inorganic fluorinated anions accounted for more than 44% of the EF and in some cases closed the mass balance. Therefore, these inorganic fluorinated anions should be monitored during EOF or EF analysis.

The presence of BF_4_^–^ and PF_6_^–^ seems to be source specific, as our data showed no detectable levels in some drinking water samples from Sweden, Denmark, and Norway. The reported concentrations of BF_4_^–^ and PF_6_^–^ did not represent the actual concentrations in drinking water and did not give any information whether they are from ionic liquids or electrolyte salts. The toxicity of BF_4_^–^ and PF_6_^–^ was found to be associated with the counter cations when applied in ionic liquids.^31^ Therefore, it is also important to evaluate and understand the levels of cations together with anions in drinking water to assess the potential health impact through drinking water in future studies.

Furthermore, very mobile and very polar PFAS and their homologues (if any) were excluded in this study due to the poor retention on the reversed column using UPLC-QTOF/MS. These compounds might also contribute to the unexplained fractions. However, measurement of these very mobile compounds is still challenging. Although SFC-HRMS has been proved to show good performance in screening small molecules,^30^ it is not widely used at present in different laboratories due to hardware availability constraints. The commonly applied chromatographic methods mainly aimed at less polar PFAS. Therefore, analytical approaches for analyzing these very mobile and very polar compounds need to be developed.

## Supporting Information Available

Detailed information about chemicals; solid phase extraction for water samples; identification of BF_4_^–^ and PF_6_^–^; mass spectrometric information for target analysis; recoveries of native PFAS in recovery samples; recoveries of IS in QC samples, real water samples, and oxidative samples; method detection limits and quantification limits; concentrations of target PFAS, BF_4_^–^, PF_6_^–^, and EF; summary of suspect screening; standards/guidelines for PFAS in drinking water; PEQ based on RPFs and figures illustrating sampling locations; the analysis of BF_4_^–^ and PF_6_^–^ using three columns; target PFAS concentrations and PEQ of tap water (PDF)
