# Supplementary material for: Further Insight into Extractable (Organo)fluorine Mass Balance Analysis of Tap Water from Shanghai, China
Source: Environ Sci Technol. 2023 Sep 15;57(38):14330–9. doi: 10.1021/acs.est.3c02718 (PMC10537424; doi:10.1021/acs.est.3c02718)
Supplement: Supplementary file 1 — es3c02718_si_001.pdf [file es3c02718_si_001.pdf]

Supporting information

Further insight into extractable (organo)fluorine mass balance analysis of tap water from Shanghai, China

Enmiao Jiao[1,2], Pontus Larsson[3], Qi Wang[4], Zhiliang Zhu[1,2], Daqiang Yin[1,2], Anna Kärrman[3], Patrick van Hees[3,5], Patrik Karlsson[5], Yanling Qiu[1,2,*], Leo W.Y. Yeung[3,*]

*Correspondence authors:

ylqiu@tongji.edu.cn;

Leo.Yeung@oru.se.

[1] Key Laboratory of Yangtze River Water Environment, College of Environmental Science and Engineering, Tongji University, Shanghai 200092, China.

[2] Shanghai Institute of Pollution Control and Ecological Security, Shanghai 200092, China

[3] Man-Technology-Environment Research Centre (MTM), School of Science and Technology, Örebro University, SE-70182 Örebro, Sweden.

[4] State Key Laboratory of Marine Pollution, City University of Hong Kong, Hong Kong 999077, China

[5] Eurofins Food & Feed Testing Sweden AB, Sjöhagsgatan 3, SE-531 40 Lidköping, Sweden

Table of content

Chemicals 3

Figure 1 Sampling sites of tap water (n=39) in Shanghai, China..4

Solid phase extraction (SPE) for water samples 5

Figure 2 Extraction workflow for target analysis (A) and extractable fluorine mass balance analysis concept (B)..6

Mass balance analysis 7

Identification of BF_4^- and PF_6^- 8

Table 1 The identification of BF_4^- and PF_6^- 8

Figure 3 Identification of BF_4^- and PF_6^- using Torous DIOL column 8

Figure 4 Identification of BF_4^- and PF_6^- using Viridis BEH Column...................................9

Figure 5 Identification of BF_4^- and PF_6^- using Viridis HSS C18 SB Column 9

Table 2 Mass spectrometric information for target analysis 10

Table 3 Recoveries of native PFAS in recovery samples (for ultra-short PFAS*, n=3 (without washing of ultrapure water with 0.01% NH4OH); for the other PFAS, n=7 (with washing of ultrapure water with 0.01%NH4OH)) 12

Table 4 Reproducibility (batch-to-batch variation) of PFAS (n=7) and internal standard (IS) recoveries in QC samples (homogeneously mixed water samples) 13

Table 5 Recoveries of IS (spiked before extractions) in real water samples (n=39)............14

Table 6 Recoveries of IS (spiked before extractions) in oxidative conversion (n=9) 15

Table 7 MDL and MQL for target analysis 16

Table 8 Concentrations of PFAS in tap water samples (ng/L, recovery-corrected for target analysis)..17

Table 9 Concentrations of EF, target PFAS and two inorganic fluorinated anions expressed in F equivalents (ngF /L) 18

Table 10 Summary of identified PFAS through suspect screening with a confidence level of 3 or above in pooled tap water samples (n=4) 19

Table 11 Standards/guidelines for PFAS in drinking water..21

Table 12 The average concentrations of PFAS in tap water from four reservoirs (A, B, C and D) and the resulting perfluorooctanoic acid equivalent (PEQ) based on RPFs.............22

Figure 6 Individual PFAS concentrations (ultra-short PFAS excluded) in tap water from different sources (A, B, C and D) 23

Figure 7 PEQ (ng/L) of tap water from four reservoirs (A, B, C and D) (line: the range of PEQ) 24

Chemicals

The majority of native standards including perfluoroalkyl carboxylic acids (PFCAs; C4-C14, C16 and C18), perfluoroalkyl sulfonic acids (PFSAs; C3-C10 and C12), fluorotelomer sulfonic acids (FTSAs; 4:2, 6:2 and 8:2), chlorinated polyfluorinated ether sulfonic acids (6:2 Cl-PFESA, 8:2 Cl-PFESA), perfluoroethylcyclohexane sulfonic acid (PFECHS), perfluorooctane sulfonamide (PFOSA) and isotope-labelled internal standards (except $^{13}C_2$-TFA) were purchased from Wellington Laboratories (Guelph, ON, Canada). The rest of the compounds were trifluoroacetic acid (TFA, purchased from Sigma-Aldrich, Munich, Germany), perfluoropropanoic acid (PFPrA, purchased from Sigma-Aldrich, Oakville, ON, Canada), trifluoromethane sulfonic acid (TFMS, purchased from Sigma-Aldrich, Stockholm, Sweden) and isotope-labelled TFA ($^{13}C_2$-TFA, purchased from Toronto Research Chemicals Inc, Toronto, Canada).

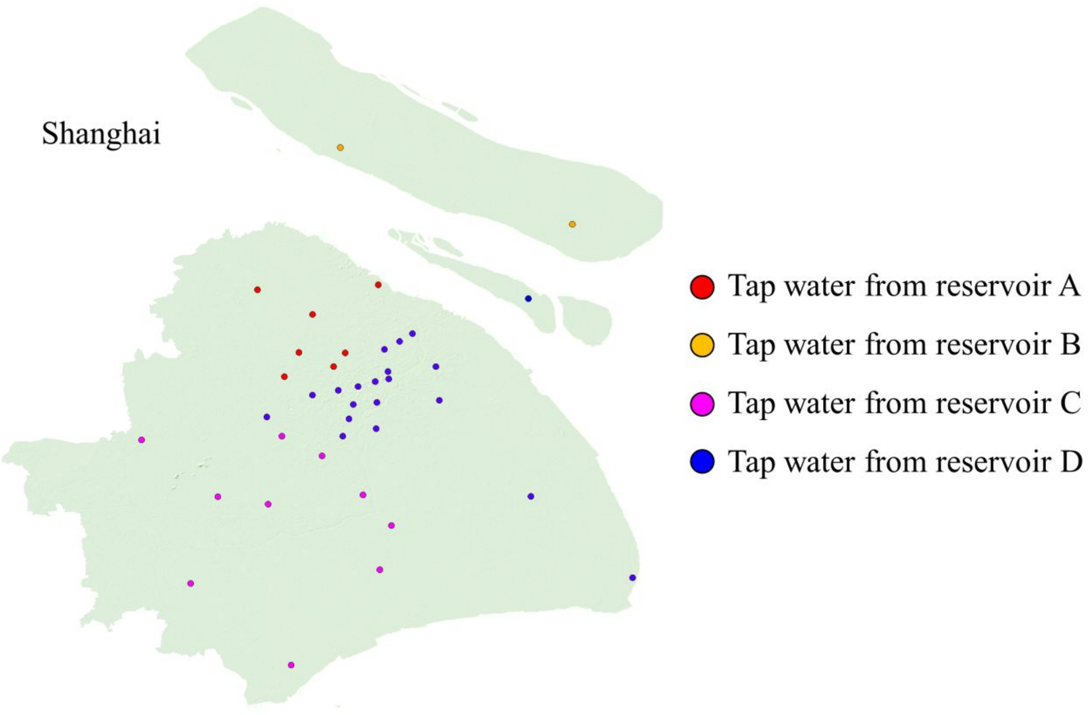

Figure 1 Sampling sites of tap water (n=39) in Shanghai, China

Solid phase extraction (SPE) for water samples

Solid phase extraction (SPE) method was performed to extract (organo)fluorine including PFAS in the tap water samples. All samples were extracted in triplicate. Replicate 1 (500 mL) was spiked with internal standards (IS) before extraction and was used for target analysis (except ultra-short PFAS). Due to low recovery of TFA (less than 5%, on average 2%) observed during method optimization, another 50 mL of water samples (replicate 2) was spiked with IS before extraction and was used for target analysis of ultra-short PFAS. Replicate 3 (1 L) was extracted without spiking any IS before extraction and was used for EF/EOF analysis.

Replicate 1 (500 mL) was spiked with IS (4 ng for IS TFA and 2 ng for other IS) before extraction, replicate 3 (1 L) was extracted without spiking any IS before extraction. The two replicates were then extracted in the same protocol using Oasis WAX cartridges (Waters 150 mg, 6 mL, 30 μm). In brief, the cartridges were first conditioned by 4 mL of methanol with 0.1% NH_4OH, 4 mL of methanol and 4 mL of ultrapure water. 20 mL of ultrapure water with 0.01% NH4OH, 30 mL of ultrapure water, 4 mL of an ammonium acetate buffer solution (pH=4) and 4 mL of ultrapure water with 20% MeOH were used as the washing step when the loading was finished. After drying under vacuum for 30 min, the cartridges were eluted with 4 mL of methanol with 0.1% NH_4OH. The extracts were then evaporated under nitrogen gas and recovery standards (RS) were spiked in the extracts of replicate 1 prior to instrumental analysis.

In order to improve TFA recovery, 50 mL of water samples (replicate 2) was extracted for quantification of ultra-short PFAS. The extraction protocol of replicate 2 followed similar procedures as replicate 1 except for skipping the washing step of ultrapure water with 0.01% NH_4OH, because the reason for low recovery for TFA was due to the washing step aiming at removing inorganic fluoride that might have enriched onto the SPE cartridge.

In addition, individual tap water samples from the same reservoir were grouped into one pool by taking equal volumes to a final total volume of 1 L. Thus, 39 individual tap water samples were grouped into four pools to represent water samples from the 4 reservoirs. They were extracted in the same way as replicate 2 and the extracts were further used for oxidative conversion and suspect screening.

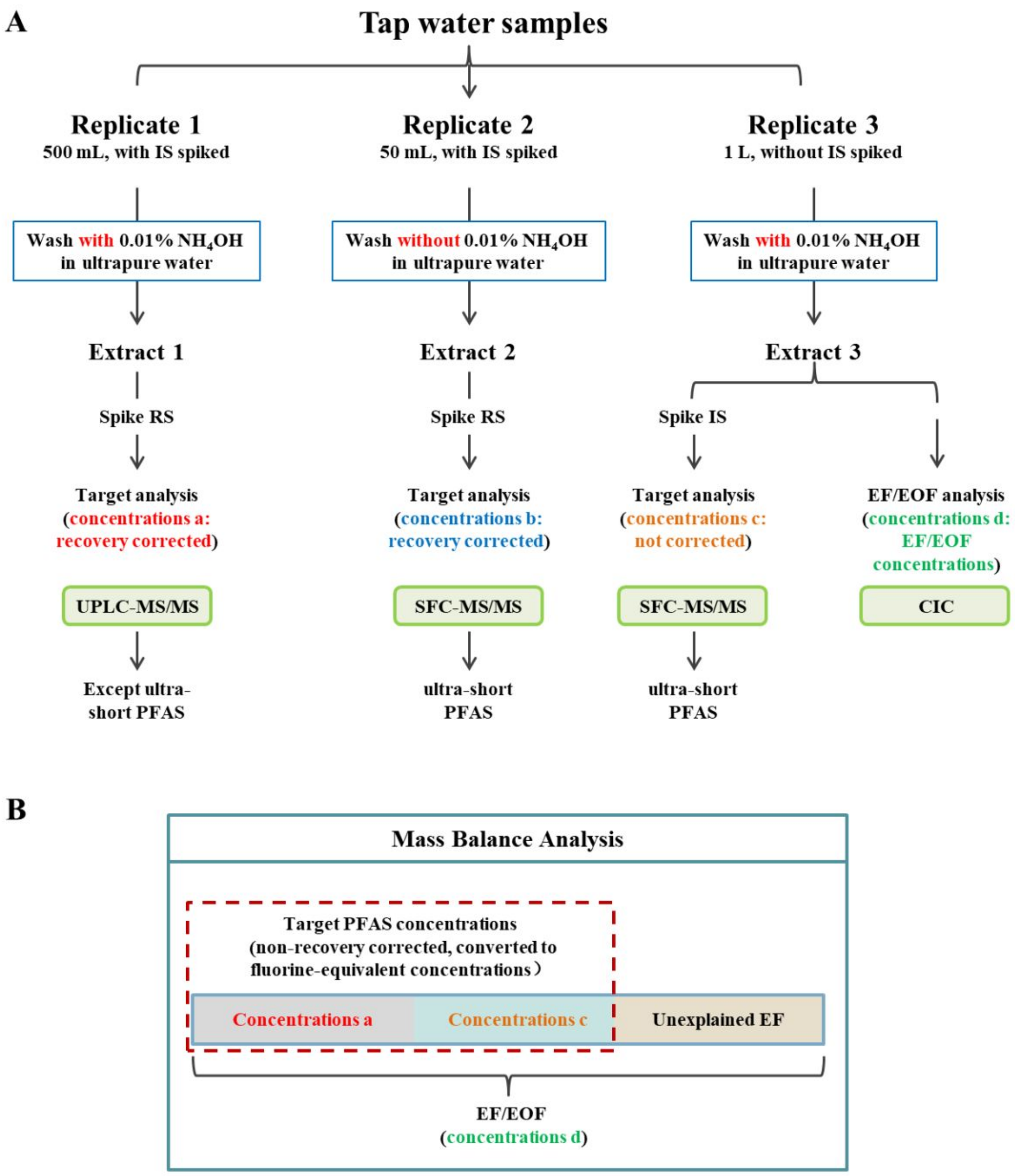

Figure 2 Extraction workflow for target analysis (A) and extractable fluorine mass balance analysis concept (B)

Mass balance analysis

Since analysis of target PFAS (except for ultra-short PFAS) and EF followed the same extraction procedures, the measured concentrations of target compounds (except for ultra-short PFAS) in EF/EOF fractions were the concentrations of these compounds in target analysis but without correcting for recoveries. The EF/EOF fractions were spiked with isotopically labelled standards and were ran to analyze the concentrations of ultra-short PFAS. The measured concentrations of all compounds have to be converted into fluorine-equivalent concentrations by multiplying the measured concentration of the compound with the molecular mass of fluorine atom in the compound as well as the number of fluorine atom on the compound, and then divided by the molecular mass of the compound before comparing to the levels of EF/EOF. Similar calculations are provided in elsewhere.[1]

Identification of BF_4^- and PF_6^-

BF_4^- and PF_6^- were quantified based on single ion monitoring due to non-detectable fragments. Samples and standards were analyzed on SFC-MS/MS using three analytical columns of different separation principles (Torous DIOL column (3 mm x 150 mm, 1.7 μm), Viridis BEH Column (3 mm x 100 mm, 1.7 μm), and Viridis HSS C18 SB Column (3 mm x 100 mm, 1.8 μm)). The results showed the retention time in samples matched to standards in the three columns. Moreover, the isotopic patterns of BF_4^- were found to match to natural isotopic distribution ($^{11}B/(^{11}B+^{10}B)$ was around 80%) which further supported the identification of BF_4^- (Figure S2-S4 and Table S1).

Table 1 The identification of BF_4^- and PF_6^-

Column	Type	BF_4^-		PF_6^-
		Rention time (min)	Isotope pattern (%) ($^{11}BF_4/(^{11}BF_4+^{10}BF_4)$)	Rention time (min)
DIOL	STD	5.54	79.7	6.16
	Sample	5.55	78.2	6.16
BEH	STD	2.60	81.0	2.73
	Sample	2.61	80.0	2.74
HSS C18 SB	STD	2.41	80.5	2.47
	Sample	2.43	80.9	2.49

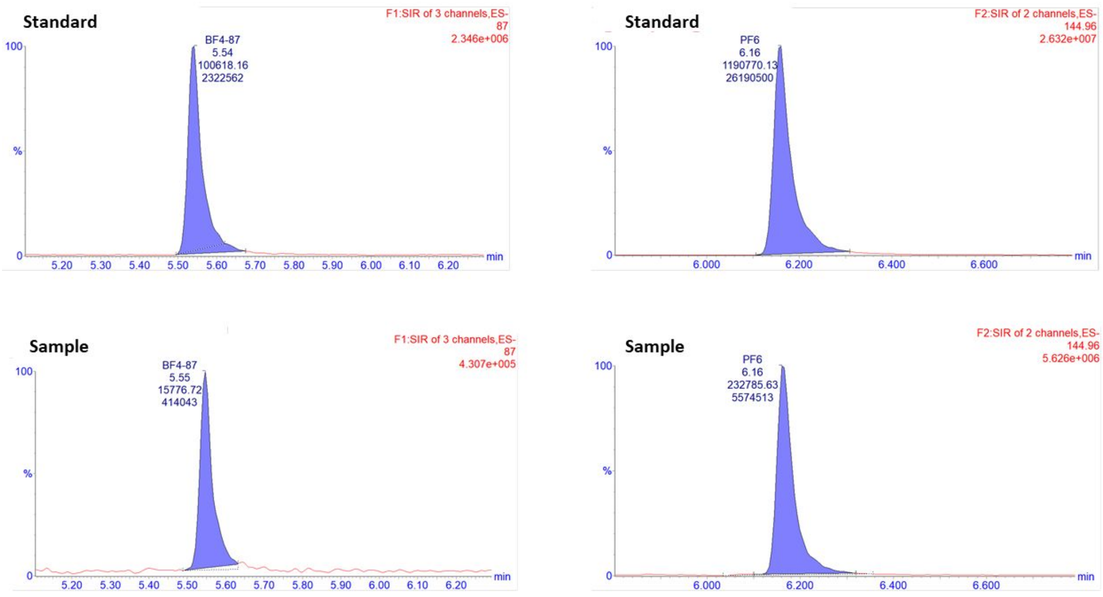

Figure 3 Identification of BF_4^- and PF_6^- using Torous DIOL column

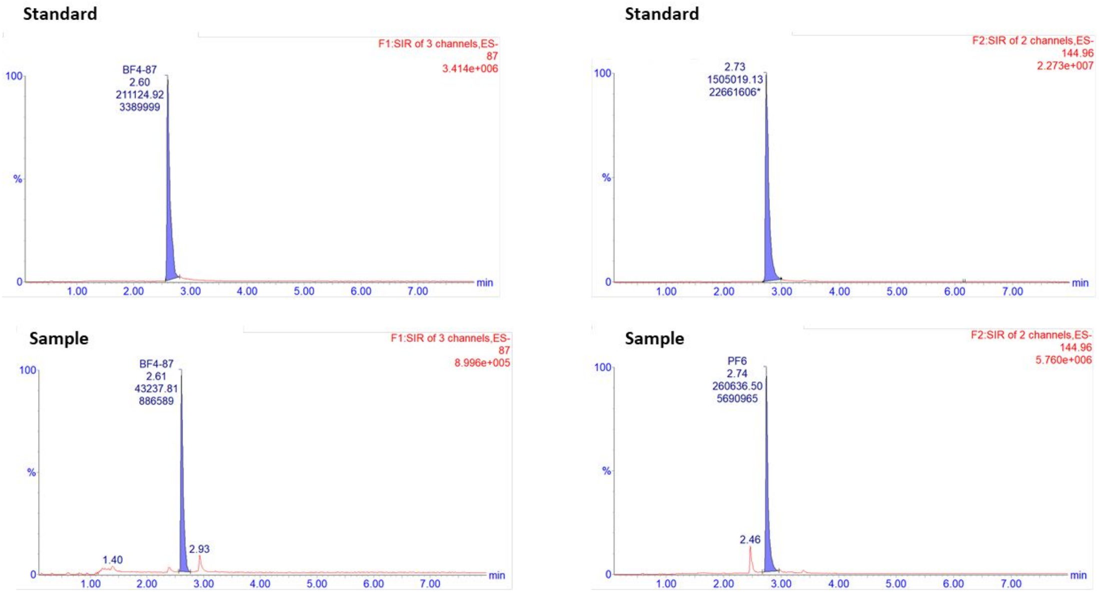

Figure 4 Identification of BF_4^- and PF_6^- using Viridis BEH Column

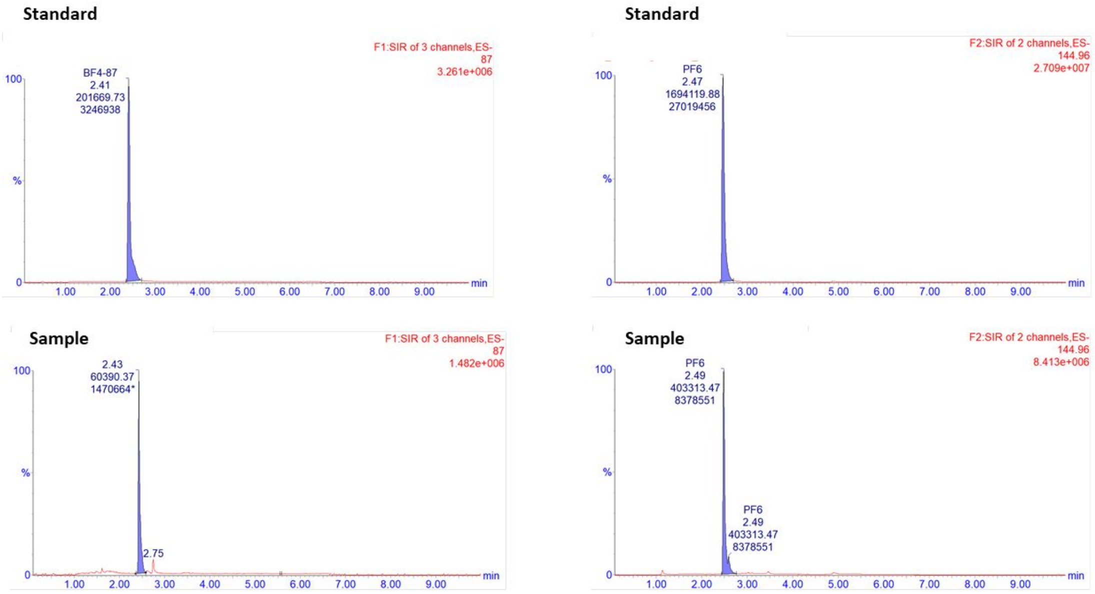

Figure 5 Identification of BF_4^- and PF_6^- using Viridis HSS C18 SB Column

Table 2 Mass spectrometric information for target analysis

Class	Abbreviation	Separation instrument	Quantification ion (m/z)	Qualification ion (m/z)	Internal standard
PFCAs	TFA	SFC	112.9/68.96	/	$^{13}C_2$-TFA
	PFPrA	SFC	162.97/118.9	/	$^{13}C_4$-PFBA
	PFBA	UPLC	212.97/169	/	$^{13}C_4$-PFBA
	PFPeA	UPLC	262.97/219	/	$^{13}C_3$-PFPeA
	PFHxA	UPLC	312.97/269	312.97/118.95	$^{13}C_2$-PFHxA
	PFHpA	UPLC	362.97/319	362.97/168.97	$^{13}C_4$-PFHpA
	PFOA	UPLC	412.97/369	412.97/168.97	$^{13}C_4$-PFOA
	PFNA	UPLC	462.99/419	462.99/219	$^{13}C_5$-PFNA
	PFDA	UPLC	512.97/469	512.97/219	$^{13}C_2$-PFDA
	PFUnDA	UPLC	562.97/519	562.97/268.99	$^{13}C_2$-PFUnDA
	PFDoDA	UPLC	612.97/569	612.97/168.96	$^{13}C_2$-PFDoDA
	PFTrDA	UPLC	662.9/619	662.9/168.96	$^{13}C_2$-PFDoDA
	PFTeDA	UPLC	712.9/669	712.9/168.97	$^{13}C_2$-PFTDA
	PFHxDA	UPLC	812.9/769	812.9/168.96	$^{13}C_2$-PFHxDA
	PFOcDA	UPLC	912.9/869	912.9/168.96	$^{13}C_2$-PFHxDA
PFSAs	TFMS	SFC	149.12/79.91	149.12/98.95	$^{13}C_3$-PFBS
	PFEtS	SFC	198.8/79.8	198.8/98.9	$^{13}C_3$-PFBS
	PFPrS	SFC	248.9/79.9	248.9/98.9	$^{13}C_3$-PFBS
	PFBS	UPLC	298.9/98.9	298.9/79.96	$^{13}C_3$-PFBS
	PFPeS	UPLC	348.9/98.96	348.9/79.96	$^{18}O_2$-PFHxS
	PFHxS	UPLC	398.9/98.9	398.9/79.96	$^{18}O_2$-PFHxS
	PFHpS	UPLC	448.97/98.9	448.97/79.96	$^{13}C_4$-PFOS
	PFOS	UPLC<br>UPLC	498.97/98.9	498.97/79.96<br>498.97/169.03	$^{13}C_4$-PFOS
	PFNS	UPLC	548.9/98.96	548.9/79.96	$^{13}C_4$-PFOS
	PFDS	UPLC	598.97/98.9	598.97/79.96	$^{13}C_4$-PFOS
	PFDoDS	UPLC	698.9/98.9	698.9/79.96	$^{13}C_4$-PFOS

	4:2 FTSA	UPLC	327/307	327/81	$^{13}C_2$-6:2 FTSA
FTSAs	6:2 FTSA	UPLC	427/407	427/81	$^{13}C_2$-6:2 FTSA
	8:2 FTSA	UPLC	527/507	527/81	$^{13}C_2$-8:2 FTSA
	ADONA	UPLC	376.97/250.8	376.97/84.69	$^{18}O_2$-PFHxS
	HFPO-DA	UPLC	284.92/168.72	284.92/184.80	$^{13}C_3$-HFPO-DA
Other PFAS	8:2 Cl-PFESA	UPLC	630.904/450.98	630.904/83.027	$^{13}C_4$-PFOS
	6:2 Cl-PFESA	UPLC	530.904/350.98	530.904/83.027	$^{13}C_4$-PFOS
	FOSA	UPLC	497.9/78	497.9/168.9	$^{13}C_8$-FOSA
	PFECHS	UPLC	460.84/380.9	460.84/98.88	$^{13}C_4$-PFOA

Table 3 Recoveries of native PFAS in recovery samples (for ultra-short PFAS*, n=3 (without washing of ultrapure water with 0.01% NH4OH); for the other PFAS, n=7 (with washing of ultrapure water with 0.01%NH4OH))

Compounds	Average Recovery	RSD	Compounds	Average Recovery	RSD	Compounds	Average Recovery	RSD
PFBA	96%	6%	PFOcDA	88%	26%	FOSA	96%	6%
PFPeA	97%	7%	HFPO-DA	92%	7%	4:2 FTSA	102%	24%
PFHxA	96%	4%	ADONA	96%	6%	6:2 FTSA	100%	9%
PFHpA	94%	8%	PFBS	97%	3%	8:2 FTSA	101%	14%
PFOA	96%	6%	PFPeS	100%	11%	F-53B	70%	9%
PFNA	93%	10%	PFHxS	99%	6%	11ClPF3OUdS	51%	46%
PFDA	94%	7%	PFHpS	119%	15%	TFA*	92%	20%
PFUnDA	94%	7%	PFOS	97%	9%	PFPrA*	95%	6%
PFDoDA	95%	6%	PFNS	73%	11%	TFMS*	96%	7%
PFTrDA	73%	6%	PFDS	61%	13%	PFEtS*	98%	6%
PFTDA	91%	8%	PFDoDS	33%	33%	PFPrS*	97%	3%
PFHxDA	94%	16%	PFECHS	100%	14%			

Table 4 Reproducibility (batch-to-batch variation) of PFAS (n=7) and internal standard (IS) recoveries in QC samples (homogeneously mixed water samples)

Compounds	Average amount (pg)	RSD	Compounds	Average Recovery	RSD	Compounds	Average Recovery	RSD
PFBA	502	9%	IS PFBA	97%	7%	IS PFDoDA	66%	22%
PFPeA	165	7%	IS PFPeA	96%	6%	IS HFPO-DA	94%	9%
PFHxA	420	7%	IS PFHxA	97%	6%	IS PFBS	105%	18%
PFHpA	210	5%	IS PFHpA	103%	8%	IS PFHxS	98%	7%
PFOA	1552	8%	IS PFOA	96%	6%	IS PFOS	89%	13%
PFNA	159	12%	IS PFNA	96%	6%	IS FOSA	75%	22%
HFPO-DA	56	14%	IS PFDA	90%	12%	IS 6:2 FTSA	92%	8%
PFBS	196	11%	IS PFUnDA	88%	15%	IS 8:2 FTSA	71%	23%
PFHxS	2393	7%	IS PFTDA	44%	18%			
PFOS	474	15%						
F-53B	187	24%						

Table 5 Recoveries of IS (spiked before extractions) in real water samples (n=39)

Compounds	Average Recovery	RSD	Compounds	Average Recovery	RSD	Compounds	Average Recovery	RSD
IS PFBA	92%	6%	IS PFUnDA	69%	14%	IS PFOS	86%	8%
IS PFPeA	93%	6%	IS PFTDA	35%	35%	IS FOSA	57%	14%
IS PFHxA	94%	6%	IS PFDoDA	51%	19%	IS 6:2 FTSA	70%	22%
IS PFHpA	96%	7%	IS PFHxDA	88%	52%	IS 8:2 FTSA	45%	36%
IS PFOA	94%	7%	IS HFPO-DA	89%	7%	IS TFA	90%	11%
IS PFNA	92%	7%	IS PFBS	92%	12%			
IS PFDA	84%	10%	IS PFHxS	97%	7%			

Table 6 Recoveries of IS (spiked before extractions) in oxidative conversion (n=9)

Compounds	Average Recovery	RSD	Compounds	Average Recovery	RSD
IS TFA	27%	20%	IS PFNA	104%	5%
IS PFBA	107%	4%	IS PFDA	98%	5%
IS PFPeA	106%	6%	IS PFUnDA	86%	7%
IS PFHxA	105%	5%	IS PFBS	98%	5%
IS PFHpA	105%	8%	IS PFHxS	112%	5%
IS PFOA	104%	5%	IS PFOS	97%	8%

Table 7 MDL and MQL for target analysis

Compounds	MDL (ng/L)	MQL (ng/L)	Compounds	MDL (ng/L)	MQL (ng/L)
PFBA	0.0464-0.137	0.0250-0.397	PFHpS		0.0250
PFPeA	/ to 0.0244	0.0250-0.0419	PFOS	/ to 0.0371	0.0250-0.107
PFHxA	/ to 0.0193	0.0250-0.0401	PFNS		0.0250
PFHpA	/ to 0.00885	0.0250-0.0255	PFDS		0.0250
PFOA	0.0474-0.116	0.0505-0.217	PFDoDS		0.0250
PFNA	/ to 0.0180	0.0250-0.0456	PFECHS		0.0250
PFDA	/ to 0.0130	0.0250-0.0323	FOSA		0.0250
PFUnDA		0.0250	4:2 FTSA		0.0250
PFDoDA	/ to 0.0206	0.0250-0.0540	6:2 FTSA	/ to 0.0459	0.0250-0.123
PFTrDA		0.0250	8:2 FTSA		0.0250
PFTDA	/ to 0.0736	0.0250-0.213	6:2 Cl-PFESA		0.0250
PFHxDA	/ to 0.0556	0.0250-0.113	8:2 Cl-PFESA		0.0250
PFOcDA	/ to 0.0440	0.0250-0.116	TFA		25
HFPO-DA		0.0250	PFPrA		25
ADONA		0.0250	TFMS		25
PFBS	/ to 0.0229	0.0250-0.0559	PFEtS		25
PFPeS		0.0250	PFPrS		25
PFHxS	/ to 0.0143	0.0250-0.0314			

Table 8 Concentrations of PFAS in tap water samples (ng/L, recovery-corrected for target analysis)

Compounds	Tap water from source A		Tap water from source B		Tap water from source C		Tap water from source D	
	Min	Max	Min	Max	Min	Max	Min	Max
PFBA	7.97	15.1	8.12	9.84	21.6	26.1	8.93	14.1
PFPeA	1.05	1.38	0.403	1.53	5.90	7.66	0.934	1.46
PFHxA	6.11	12.2	0.199	2.78	14.4	17.8	3.53	7.97
PFHpA	0.301	0.967	0.0307	0.968	5.97	8.54	0.366	1.46
PFOA	2.05	13.3	0.473	18.4	26.2	42.7	2.57	19.0
PFNA	0.0408	0.586	<MQL	0.540	2.67	5.79	0.0523	0.665
PFDA	n.d.	0.186	<MQL	0.0814	1.24	2.84	0.0297	0.136
PFUnDA	n.d.	0.0638	n.d.	0.0417	0.337	1.08	n.d.	0.0584
PFDoDA	n.d.	n.d.	n.d.	n.d.	<MQL	0.115	n.d.	<MQL
PFTrDA	n.d.	<MQL	n.d.	<MQL	n.d.	0.0283	n.d.	<MQL
PFHxDA	<MQL	0.0509	0.0425	0.0628	n.d.	0.0544	n.d.	0.0715
HFPO-DA	0.284	0.415	0.119	0.393	2.28	2.84	0.233	0.526
PFBS	1.53	3.21	0.141	2.73	9.93	14.5	1.65	5.98
PFPeS	<MQL	0.0781	<MQL	0.0703	0.0437	0.101	0.0289	0.149
PFHxS	0.0590	0.467	<MQL	0.492	3.48	13.6	0.0611	0.947
PFHpS	n.d.	0.0889	n.d.	0.0276	0.0490	0.238	n.d.	0.0657
PFOS	n.d.	1.53	<MQL	0.798	2.27	5.98	0.0429	1.12
FOSA	n.d.	0.0696	n.d.	n.d.	n.d.	n.d.	n.d.	0.104
6:2 FTSA	n.d.	0.494	<MQL	0.172	0.0364	0.394	n.d.	0.329
F-53B	n.d.	0.258	n.d.	0.0799	0.546	2.40	n.d.	0.160
TFA	1.71e3	2.07e3	1.56e3	1.82e3	6.23e3	8.03e3	1.35e3	2.01e3
PFPrA	12.9	18.9	14.7	15.3	32.9	50.6	12.0	18.0
TFMS	62.0	102.2	52.5	100.0	217.4	277.1	60.9	97.7

n.d.: <MDL.

Table 9 Concentrations of EF, target PFAS and two inorganic fluorinated anions expressed in F equivalents (ngF /L)

Compounds	Tap water from source A		Tap water from source B		Tap water from source C		Tap water from source D	
	Min	Max	Min	Max	Min	Max	Min	Max
Sum target PFAS	40.8	116	51.2	57.3	125	316	33.3	179
BF_4^-	35.1	181	69.6	73.7	23.3	131	9.32	195
PF_6^-	209	310	92.7	115	333	391	100	166
EF	293	486	160	238	600	928	179	554

Table 10 Summary of identified PFAS through suspect screening with a confidence level of 3 or above in pooled tap water samples (n=4)

	Parent ions									Fragments			
	Name	Theoretical MS	Observed MS	Error (ppm)	Molecular formula	RT (min)	CLs	PFAS used for semi-quantification	Concentrations (ng/L)	Theoretical MS	Observed MS	Error (ppm)	Molecular formula
NTf2	NTf2	279.9178	279.9171 to 279.9176	-2.5 to -0.7	C2HF6O4NS2	3.37 to 3.39	2	PFBS	0.04-0.64	77.96627	77.9626-77.9653	-47.6 to -12.7	NSO2-
										146.96135	146.9601 to 146.9603	-8.6 to -6.9	CF3SO2N-
OBS	OBS	602.9564	602.9572	1.3	C15H5F17O4S	10.47	2	PFOS	0.006	108.02168	108.0212	-4.3	C6H4O2-
										171.98358	171.9829	-3.9	C6H4O4S-
										348.97749	348.9853	22.4	C11H4O3F7S-
										464.96601	464.9778	25.2	C13H4O4F11S-
H-PFESA	2:2 H-PFESA	296.9473	296.9469	1.45	C4H2F8O4S	3.95	2	PFBS	0.002	96.99067	96.9904	-2.8	C2OF3-
										116.9969	116.9981	10.3	C2HOF4-
	6:2 H-PFESA	496.9346	496.9342	0.72	C8H2F16O4S	8.46	2	PFOS	0.05	230.98618	230.98516	-4.4	C5F9-
										296.9779	296.97691	-3.3	C6OF11-
										316.98413	316.98348	-2.1	C6HOF12-
PFSM	FBSA	297.959	297.9580 to 297.9582	-3.3 to -2.6	C4H2F9O2NS	6.59 to 6.61	2	PFHxS	0.004-0.008	77.96552	77.9652 to 77.9655	-4.1 to 0	NO2S-
	FBSAA	355.9645	355.9631 to 355.9635	-3.8 to -2.7	C6H4F9O4NS	6.24 to 6.26	3	PFHxA	0.003-0.006	218.98618	218.9863 to 218.9883	0.7 to 9.5	C4F9-
										297.95898	297.9560 to 297.9597	-10.1 to 2.3	C4HNO2F9S-
	MeFBSAA	369.9801	369.9790 to 369.9792	-3.0 to -2.5	C7H6F9O4NS	8.69 to 8.70	3	PFHpA	0.19-0.31	82.96085	82.9621	16	O2FS-
										218.98618	218.9855 to 218.9857	-2.9 to -2.1	C4F9-
H-PFCA	H-PFDA	494.9695	494.9682 to 496.9692	-2.5 to -0.5	C10H2F18O2	8.69 to 8.70	3	PFDA	0.034-0.11	101.0020	101.0016 to 101.0019	-4.2 to -1.3	C2HF4-

118.9926	118.9919 to 118.9922	-5.3 to -2.9	C2F5-
168.9894	168.9866 to 168.9885	-16.2 to -5.0	C3F7-
218.9862	218.9856 to 218.9857	-2.5 to -2.1	C4F9-
368.9766	368.9754 to 368.9759	-3.2 to -1.9	C7F15-

*: MS2 data was manually annotated;
Concentrations were semi-quantified.

Table 11 Standards/guidelines for PFAS in drinking water

Location	Agency	Year	Guideline Value （ng/L）	Type (Standard/Guideline)	Promulgated
China	-	2022	PFOA (80), PFOS (40)	Standard	Yes
USA	USEPA	2016	PFOS+PFOA (70)	Lifetime Health Advisory	-
California, USA	The State Water Resources Control Board	2021	PFOA (5.1), PFOS (6.5)	Notification Levels	-
Vermont, USA	Department of Health	2020	PFOA+PFOS+PFHxS+PFHpA+PFNA (20)	Maximum Contaminant Levels	Yes
Massachusetts, USA	Department of Environmental Protection	2019	PFOA+PFOS+PFNA+PFHxS+PFHpA+PFDA (20)	Drinking Water Values	-
New York, USA	Department of Health	2020	PFOA (10), PFOS (10)	Maximum Contaminant Levels	Yes
European Union	European Commission	2021	PFAS total (500)	Proposed Drinking Water Directive	Yes
Denmark	EPA	2021	PFOA+PFOS+PFNA+PFHxS (2)	Health Based Guidance	-
Sweden	Swedish Food Agency	2022	PFOA+PFNA+PFOS+PFHxS (4)	Limit values	-
			PFBA+PFPeA+PFHxA+PFHpA+PFOA+PFNA+PFDA+PFUnDA+PFDoDA+PFTrDA+PFBS+PFPeS+PFHxS+PFHpS+PFOS+PFNS+PFDS+PFUnDS+PFDoDS+PFTrDS+6:2FTS (100)<br>(* This value is not included in this study since some PFAS (PFUnDS+PFDoDS+PFTrDS) were not included in this study.)		-

*: Promulgated: Yes means values they have been finalized into law or are referenced in supporting law.

Table 12 The average concentrations of PFAS in tap water from four reservoirs (A, B, C and D) and the resulting perfluorooctanoic acid equivalent (PEQ) based on RPFs

PFAS	RPFs	Tap water from reservoir A		Tap water from reservoir B		Tap water from reservoir C		Tap water from reservoir D	
		Average Concentrations (ng/L)	PEQ* (ng/L)	Average Concentrations (ng/L)	PEQ* (ng/L)	Average Concentrations (ng/L)	PEQ* (ng/L)	Average Concentrations (ng/L)	PEQ* (ng/L)
PFBA	0.05	12.51	0.63	8.98	0.45	23.98	1.2	10.64	0.53
PFPeA	0.01≤RPF≤0.05	1.19	0.01≤PEQ≤0.06	0.97	0.01≤PEQ≤0.05	6.79	0.07≤PEQ≤0.34	1.26	0.01≤PEQ≤0.06
PFHxA	0.01	8.35	0.08	1.49	0.01	16.00	0.16	5.73	0.06
PFHpA	0.01≤RPF≤1	0.48	0.005≤PEQ≤0.48	0.50	0.01≤PEQ≤0.50	6.91	0.07≤PEQ≤6.91	1.10	0.01≤PEQ≤1.10
PFOA	1	5.15	5.15	9.44	9.44	33.67	33.67	13.58	13.58
PFNA	10	0.19	1.93	0.28	2.82	4.19	41.93	0.47	4.68
PFDA	4≤RPF≤10	0.07	0.29≤PEQ≤0.72	0.05	0.21≤PEQ≤0.53	2.04	8.15≤PEQ≤20.36	0.09	0.37≤PEQ≤0.92
PFUnDA	4	0.04	0.15	0.04	0.17	0.75	3	0.04	0.14
PFDoDA	3	-	-		-	0.06	0.19	0.025	0.08
PFTrDA	0.3≤RPF≤3	0.025	0.01≤PEQ≤0.08	0.025	0.01≤PEQ≤0.08	0.027	0.01≤PEQ≤0.08	0.025	0.01≤PEQ≤0.08
PFHxDA	0.02	0.09	1.80E-03	0.05	0.001	0.05	0.001	0.06	0.001
HFPO-DA	0.02	0.34	0.01	0.26	0.005	2.56	0.05	0.34	0.01
PFBS	0.001	2.09	0.002	1.44	0.001	11.47	0.01	4.34	0.004
PFPeS	0.001≤RPF≤0.6	0.04	4.12E-5≤PEQ≤0.02	0.05	4.77E-5≤PEQ≤0.03	0.07	6.51E-5≤PEQ≤0.04	0.09	8.90E-5≤PEQ≤0.05
PFHxS	0.6	0.25	0.15	0.26	0.16	5.64	3.38	0.55	0.33
PFHpS	0.6≤RPF≤2	0.06	0.04≤PEQ≤0.13	0.03	0.02≤PEQ≤0.05	0.15	0.09≤PEQ≤0.30	0.04	0.02≤PEQ≤0.08
PFOS	2	0.52	1.03	0.45	0.91	3.87	7.75	0.75	1.51
Sum PEQ			9.49≤PEQ≤10.63		14.21≤PEQ≤15.13		99.73≤PEQ≤119.38		21.35≤PEQ≤23.22

* If an individual PFAS was not detected in some samples, these samples were excluded when estimating PEQ of this individual PFAS. Concentrations below MQL were set as MQL.

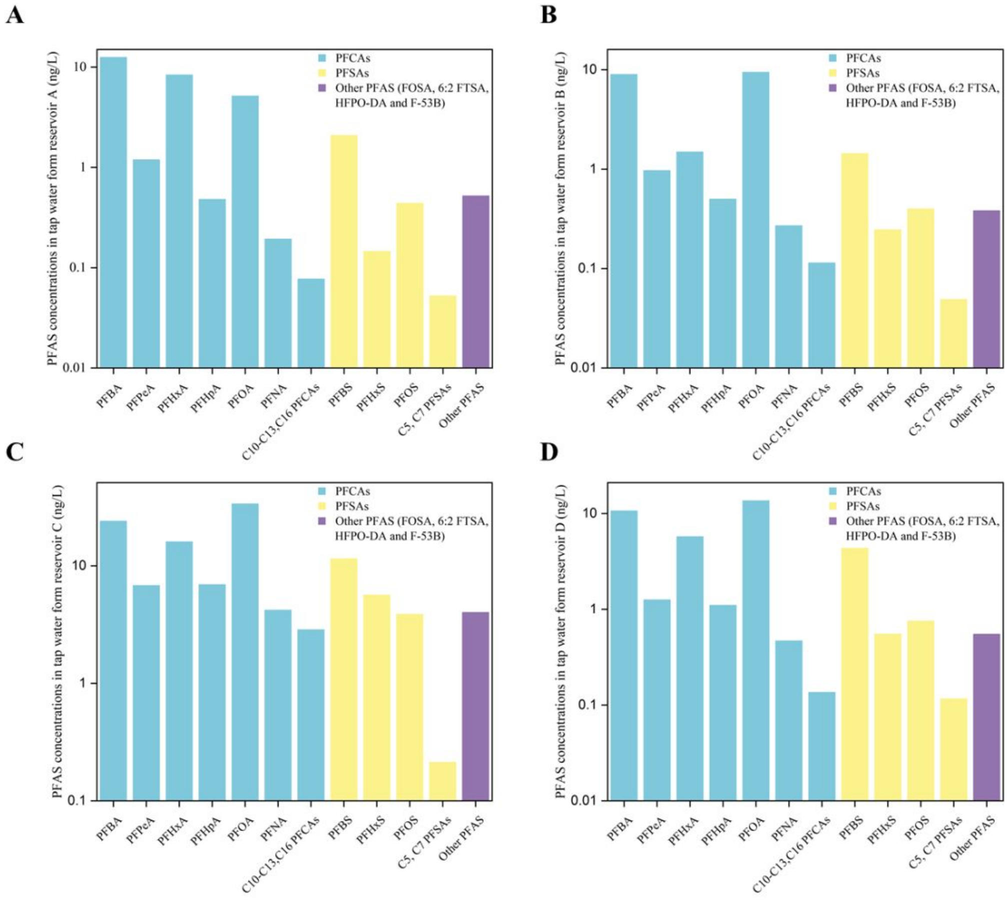

Figure 6 Individual PFAS concentrations (ultra-short PFAS excluded) in tap water from different sources (A, B, C and D)

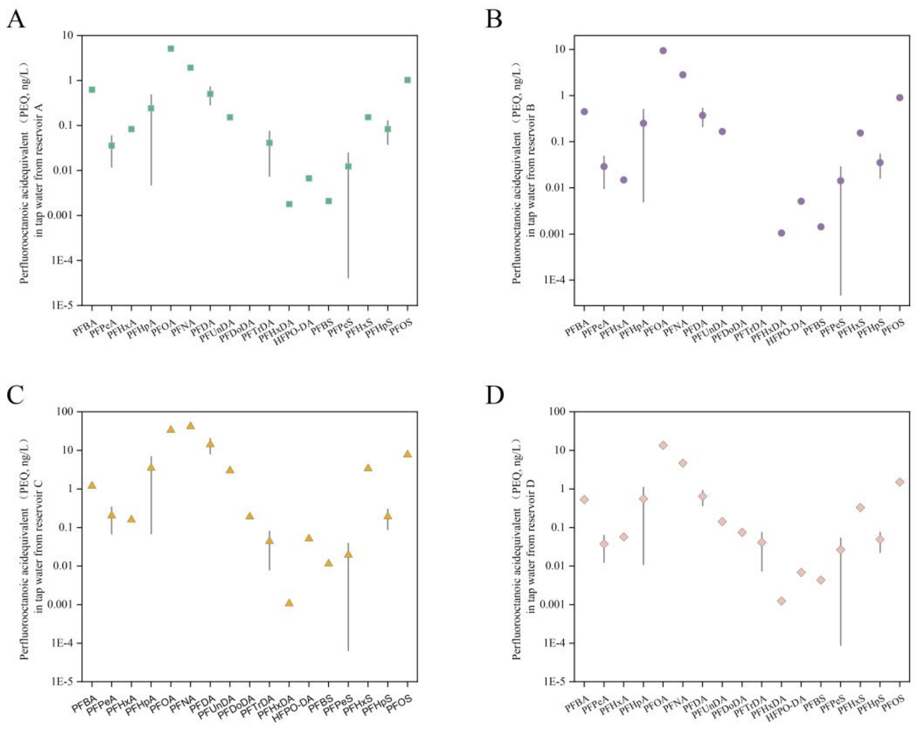

Figure 7 PEQ (ng/L) of tap water from four reservoirs (A, B, C and D) (line: the range of PEQ)

1. Miyake, Y.; Yamashita, N.; Rostkowski, P.; So, M. K.; Taniyasu, S.; Lam, P. K. S.; Kannan, K. Determination of trace levels of total fluorine in water using combustion ion chromatography for fluorine: A mass balance approach to determine individual perfluorinated chemicals in water. *J. Chromatogr. A* **2007,** *1143*, (1), 98-104.
